# Targeting senescent cells for a healthier longevity: the roadmap for an era of global aging

**DOI:** 10.1093/lifemedi/lnac030

**Published:** 2022-08-09

**Authors:** Yu Sun, Qingfeng Li, James L Kirkland

**Affiliations:** CAS Key Laboratory of Tissue Microenvironment and Tumor, Shanghai Institute of Nutrition and Health, Chinese Academy of Sciences, Shanghai 200031, China; Department of Pharmacology, Institute of Aging Medicine, Binzhou Medical University, Yantai 264003, China; Department of Medicine and VAPSHCS, University of Washington, Seattle, WA 98195, USA; Department of Plastic and Reconstructive Surgery, Shanghai Ninth People’s Hospital, Shanghai Jiao Tong University School of Medicine, Shanghai 200011, China; Department of Medicine, Mayo Clinic, Rochester, MN 55905, USA; Department of Physiology and Biomedical Engineering, Mayo Clinic, Rochester, MN 55905, USA

**Keywords:** aging, senescent cell, senescence-associated secretory phenotype, senotherapeutics, clinical trial

## Abstract

Aging is a natural but relentless process of physiological decline, leading to physical frailty, reduced ability to respond to physical stresses (resilience) and, ultimately, organismal death. Cellular senescence, a self-defensive mechanism activated in response to intrinsic stimuli and/or exogenous stress, is one of the central hallmarks of aging. Senescent cells cease to proliferate, while remaining metabolically active and secreting numerous extracellular factors, a feature known as the senescence-associated secretory phenotype. Senescence is physiologically important for embryonic development, tissue repair, and wound healing, and prevents carcinogenesis. However, chronic accumulation of persisting senescent cells contributes to a host of pathologies including age-related morbidities. By paracrine and endocrine mechanisms, senescent cells can induce inflammation locally and systemically, thereby causing tissue dysfunction, and organ degeneration. Agents including those targeting damaging components of the senescence-associated secretory phenotype or inducing apoptosis of senescent cells exhibit remarkable benefits in both preclinical models and early clinical trials for geriatric conditions. Here we summarize features of senescent cells and outline strategies holding the potential to be developed as clinical interventions. In the long run, there is an increasing demand for safe, effective, and clinically translatable senotherapeutics to address healthcare needs in current settings of global aging.

## Introduction

Aging represents a natural process characterized by progressing physiological, cellular, and molecular alterations, which together contribute to physical dysfunction, frailty, and eventually, a host of age-related diseases. Among the hallmarks of aging is cellular senescence, which engages or connects with several of the other hallmarks [1]. Cellular senescence is a stable, antiproliferative, and usually irreversible cell fate that plays an essential role in diverse pathophysiological conditions [2, 3]. The number and scope of preclinical and early phase clinical studies of aging and senescence studies have been increasing over the last years since the critical roles of senescence across a wide range of human age-associated disorders and fundamental physiological processes have been identified, with therapeutic interventions targeting senescence *in vivo* developed [4, 5]. In this article, we provide an overview of recent biomedical advances in the senescence field, where a few breakthroughs have entered the mainstream of aging biology and geriatric medicine and hold the potential to support translation into clinical interventions in the near future.

## Human lifespan, longevity, and cellular senescence

Although the natural lifespan of humans used to be ~30 years, improvements in living and working conditions, public health and sanitation, healthcare, and pharmacological treatments for acute diseases have extended this to almost 80 years in most developed countries [6]. Unfortunately, the vast majority of the world’s population has to confront chronic disorders and diseases associated with aging. As the global aging population steadily increases, age-related pathologies have become a leading healthcare issue [7].

Chronological age is considered as a major predictor for human disorders and diseases, with mounting evidence suggesting that aging itself may contribute to worldwide morbidity and mortality [7]. In most cases, chronological and biological aging is correlated, whereas the latter can be accelerated by the development or multiple chronic pathologies, leading to chronic diseases, and geriatric syndromes [8]. Therefore, it is necessary to unravel the key causes of aging, an effort that holds the potential to reduce multimorbidity globally.

**Figure 1.**
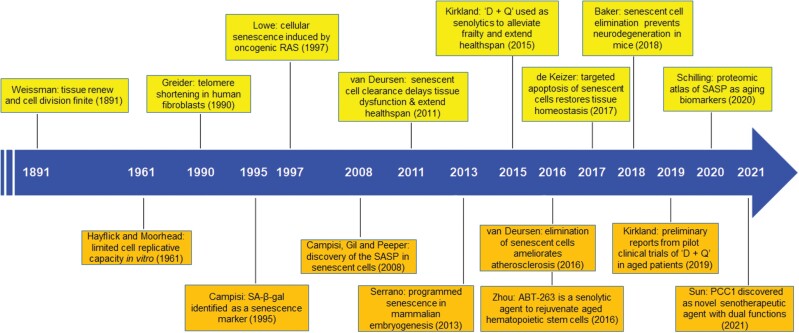
Major timelines depicting historic milestones in cellular senescence research. The first theory defining inherent limitations of tissue self-renewal, cell proliferation and organismal developmental potential was proposed in 1891 by August Weissman, interpreting the distinction between the heritable germline and the perishable soma. In 1961, Hayflick et al. Moorhead reported the finite proliferation potential of human primary fibroblasts, which can be observed after serial passaging in culture and was termed “cellular senescence.” Years afterwards, numerous scientists not only confirmed this finding, but made continued and significant efforts to reveal mechanisms underlying senescence and to identify biological properties of senescent cells. As one of the critical breakthroughs in aging biology, several groups demonstrated that clearance of senescent cells via targeted agents in animal models and, more recently, in aged humans who typically develop chronic pathologies, can delay age-related dysfunction, and alleviate age-related diseases. The key breakthroughs are individually presented in the order of the name of major scientist, a brief description of the central finding or concept, as well as the year of the work published. “D + Q,” a combination of dasatinib and quercetin.

More than a century ago, the German biologist August Weissman proposed that a worn-out tissue cannot forever renew itself, and its capacity of regrowth via cell division is not everlasting but indeed finite, defining the limited duration of life [9]. From an evolutionary perspective, healthy longevity is subject to modulation by biological forces generated by natural selection, which confers fitness in early life but may result in detrimental effects in later stages. One such force is cellular senescence, which arrests the proliferation of neoplastic cells but compromises the health of the aged by damaging tissues that harbor an increasing number of those senescent cells with a tissue-damaging secretory phenotype (SASP) [10, 11]. Despite the historic discovery by Hayflick and Moorhead in the 1960s who studied primary human cells cultured *in vitro* during serial passaging [12], the impact of cellular senescence on health has only recently been recognized with experimental findings of its implications across a range of age-related disorders in diverse mammalian species and organ systems (Fig. 1).

**Figure 2.**
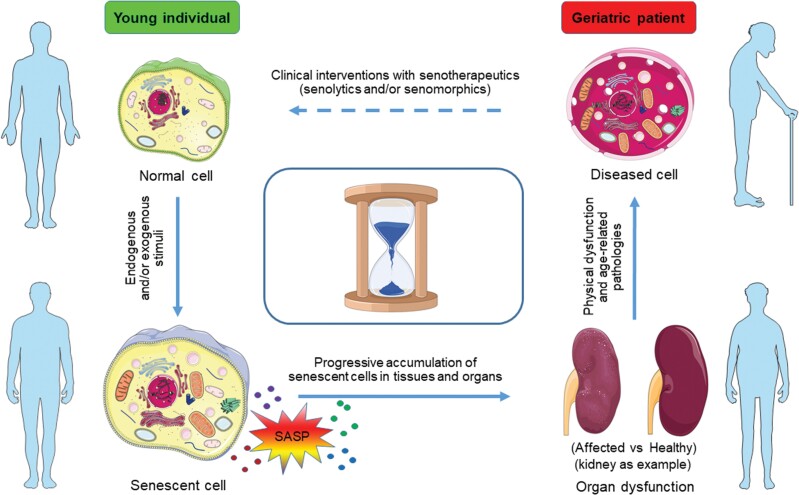
The impact of senescent cells on human aging and age-related pathologies and emerging interventions. During the lifespan senescent cells can accumulate, which secret a plethora of extracellular pro-inflammatory factors and/or other factors, collectively referred to as the SASP. Notably, some SASP components can not only reinforce cellular senescence per se, but induce passive senescence of adjacent and distant cells, together resulting in tissue remodeling and causing age-related organ dysfunction, such as nephropathy (presented as graphic symbols of the kidney, affected vs. healthy). Age-associated degeneration in several key organs contributes to organismal aging, affecting healthspan, and lifespan. As part of these recent advances, small molecule compounds that specifically target senescent cells (namely senotherapeutics, including senomorphics and senolytics) and minimize their detrimental effects *in vivo* are being intensively developed. Such agents with a potential to promote tissue rejuvenation, provide new therapeutic opportunities for intervening against multiple age-associated conditions. The clock at the center represents circadian rhythm or time relapse in the course of human aging.

The nine hallmarks of aging proposed in 2013 by López-Otín et al. are essentially not independent, but rather appear to be interdependent [1, 13]. The Unitary Theory of Fundamental Aging Mechanisms postulates that by targeting one fundamental aging process, may be possible to alleviate several or all of the others [13–15]. Cellular senescence is bidirectionally linked to the other hallmarks of aging, including epigenetic alterations, mitochondrial dysfunction, nutrient mis-sensing, stem cell exhaustion, altered cellular communication, genomic instability, telomere attrition, and loss of proteostasis [1, 15]. Given the diverse established and well-documented interactions with other hallmarks of aging, cellular senescence appears to be a central hub and appropriate target for therapeutic intervention (Fig. 2). Recent studies proved that selective elimination of senescent cells by genetic approaches and pharmacological agents (senolysis), which began to be developed even before genetic models were made [13], can significantly extend median lifespan and effectively attenuate age-associated pathologies [6, 15], inspiring the development of targeted senolytic pharmaceutics to eliminate senescent cells as a novel and potentially potent healthspan-extending modality [14].

## Cellular senescence and its fundamental properties

Characterization of senescence has been a complex, challenging, and controversial task, although there are several well-defined features of senescent cells. In response to damaging insults such as telomere attrition, chemotherapeutic agents, and ionizing radiation, senescence-associated arrest signals usually converge on the p53-p21^CIP1^ and p16^INK4a^-retinoblastoma (RB) pathways [16]. Upregulated cyclin-dependent kinase inhibitors, frequently including p21^CIP1^ and p16^INK4a^, cause blockade of the cell cycle at G1/S, which occurs upon formation of the cyclin–cyclin-dependent kinase complex and failure to inactivate the RB protein [17].

Despite the convergence of senescence-related growth arrest pathways, different inducing stressors may result in distinct senescent cell subpopulations [18, 19], a fact that may help explain the current lack of a single generally accepted marker or a gold standard to define senescence. Furthermore, a recent study suggested that cell types can differentially reprogram their transcriptome in response to even the same stimuli [20]. Distinct from well-controlled cell culture models, various stressors are able to act simultaneously both in the case of aging and in the context of pathologies in organisms, thus making it easier to understand why there is such diversity and heterogeneity among senescent cell populations *in vivo* [11].

In contrast to their proliferating counterparts, senescent cells generally exhibit increased volume and enhanced granularity, changes presumably correlated with their elevated metabolism, increased protein production, decreased autophagy, lipid accumulation, and compromised homeostasis [21]. Both lysosomes and mitochondria tend to accumulate in the cytoplasm upon cellular senescence, additional features for senescent cell identification. Specifically, increased abundance of lysosomes with a higher pH than normal cells is accompanied by enhanced activity of senescence-associated beta galactosidase (SA-β-Gal), a commonly used marker that allows quick and straightforward detection of senescent cells [22, 23]. Despite these advantages, the SA-β-Gal activity assay is not fully sensitive and specific, and is recommended to be used in concert with other markers for accurate determination of whether cells are senescent. As a form of aggregates of lysosome-associated byproducts, lipofuscins frequently accumulate in senescent cells, providing another way for evaluating senescent cells, although with limitations in specificity similar to that of the SA-β-Gal assays [24].

The mitochondria of senescent cells are increased in mass and number, have altered morphology, reduced membrane potential and enhanced reactive oxygen species (ROS) production, oxidative phosphorylation, electron transport, and oxygen consumption [25, 26]. Although there is a correlation between mitochondrial dysfunction and cellular senescence, whether senescence is a cause or consequence of mitochondrial dysfunction remains unclear [27, 28]. Depletion of mitochondrial sirtuins, a family of evolutionarily conserved proteins that are related to aging phenotypes across different species, and that can selectively inhibit certain mitochondrial functions, can trigger cellular senescence, while preserving ATP production through enhanced glycolysis [29]. In senescent cells, the cascade of ATM, Akt, and mTORC1 phosphorylation integrates signals from the DNA damage response (DDR) complex toward PGC-1β-dependent mitochondrial biogenesis, causing ROS-mediated activation of the DDR and cell cycle arrest, suggesting a link between nuclear DNA damage and mitochondrial dysfunction [30]. Interestingly, mitochondrial dysfunction-associated senescence has a unique cell-non-autonomous program that is potentially responsible for altered metabolism and aberrant adipocyte differentiation in aged animals [29]. Notably, the role of dysfunctional mitochondria extends beyond simply production of ROS in senescence induction [26, 31]. Senescent cells can have redox imbalance of NAD^+^/NADH and NADP^+^/NADPH pairs, as they appear to depend on high cytoplasmic NADH but low NADPH, in contrast to proliferating cells which have high cytoplasmic NAD^+^ and normal NADPH levels [32]. Furthermore, mitophagy deficiency-induced accumulation of damaged mitochondria is an important trigger of cellular senescence, while cellular senescence can directly dysregulate mitophagy [26].

**Figure 3.**
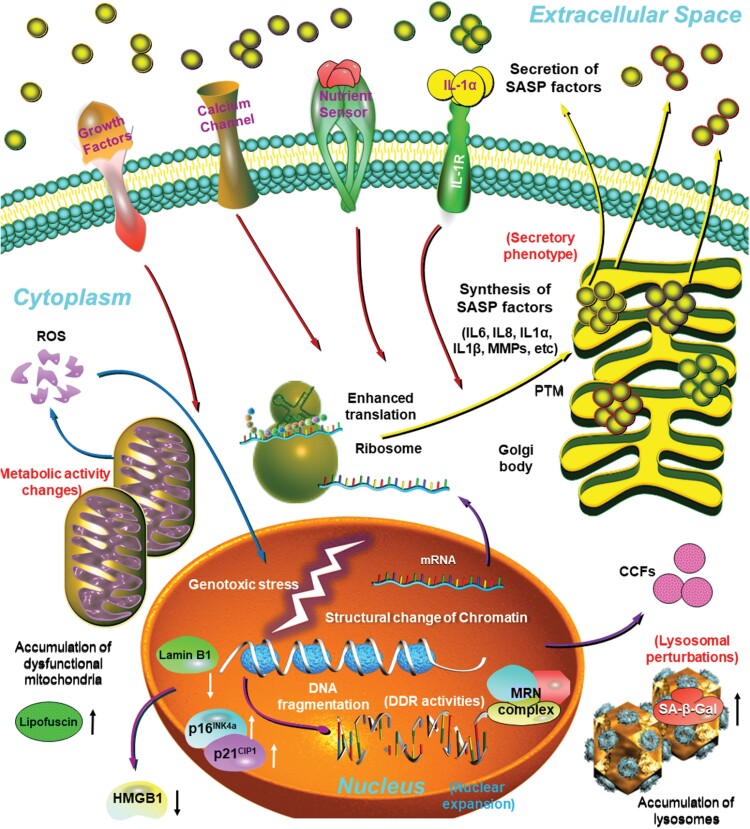
Major characteristics and distinct markers of senescent cells. In response to endogenous stimuli and/or environmental stress, proliferating cells can be forced to into a stable and usually irreversible state of cell cycle arrest, termed cellular senescence. Of note, senescent cells are typically enlarged, flattened, exhibit an irregular cell shape, and develop a secretory phenotype, the SASP, which can be pro-inflammatory and pro-fibrotic. Their nuclear integrity is compromised due to the loss of Lamin B1 and nuclear exclusion of the HMGB1, with the appearance of extranuclear DNA species, particularly CCFs. Senescent cells display increased lysosomal content and enhanced SA-β-Gal activity, with an increased number of large but dysfunctional mitochondria producing high levels of ROS. There is often an elevated level of lipofuscin in the cytoplasm, up-regulated expression of p16^INK4a^/p21^CIP1^ and aberrant histone modifications in the nucleus. Biosynthesis and extracellular release of a number of pro-inflammatory factors as part of the SASP can be a prominent hallmark or feature of senescent cells. CCF, cytoplasmic chromatin fragments; HMGB1, high mobility group box 1. Dark red arrows, signal transduction initiated upon binding of extracellular ligands to their corresponding receptors at the plasma membrane. Blue arrows, generation and transportation of ROS in the senescent cell. Purple arrows, transition within and release of molecules from the nucleus. Yellow arrows, protein translation, posttranslational process, and extracellular release of SASP factors. White and black arrows beside molecules or organelles, up- or down-regulation of expression level or biological activity of indicated molecules in the nucleus (white) or the cytoplasm (black), individually.

There are remarkable molecular changes in the nuclei of senescent cells, a feature inherently associated with DDR pathways, which result in upregulated expression of p16^INK4a^ and p21^CIP1^, enhanced activation of H2AX, the DDR regulatory histone that becomes phosphorylated and coordinates DNA repair activities upon genotoxic stress [33]. As another form of nuclear alterations, a nuclear intermediate filament protein and epigenetic modulator, lamin B1, is lost [34]. Furthermore, specific chromatin remodeling patterns termed senescence-associated heterochromatin foci (SAHFs) and decreased DNA replication can be observed, both of which can be assayed by immunofluorescence [35, 36]. In addition, genetic and epigenetic markers such as derepression of LINE-1 retrotransposons and up-regulation of the histone acetyltransferase KAT7 can be detected in nuclei upon cellular senescence [37, 38] (Fig. 3).

## Molecular regulation and pathological implications of the SASP

Reported in 2008 by Campisi’s group, the SASP has been defined as a core hallmark of senescent cells and was confirmed in the same year by Gil and Peeper groups [39–41]. In the microenvironment, the SASP can be a critical player responsible for the combined physiologic and pathologic effects of senescent cells. The SASP encodes a large array of cytokines, chemokines, growth factors, matrix metalloproteinases, microRNAs, and other noncoding nucleotides such as mitochondrial DNA, and small molecule metabolites, which are secreted directly or *via* extracellular vesicles into the microenvironmental space [3, 42–44]. The production of these molecules is usually regulated by p38MAPK, TAK1, JAK2/STAT3, p53, mTOR, NF-κB, c/EBPβ, GATA4, and Zscan4 [36, 45–49]. Increasing lines of evidence further suggest that development of the SASP is subject to multilayered modulation, including control by the cGAS-STING pathway and regulation at epigenetic levels such as remodeling in the super-enhancer cascade landscape [50–52].

The nature, composition and destructiveness or benefits of the SASP can vary substantially depending on the individual cell type that becomes senescent, and the nature of the senescence-inducing stimulus, with oncogene-induced senescence being characterized with a higher capacity of protein secretion compared with replicative or radiation-induced senescence [39]. For example, SARS-CoV-2 can make the SASP of existing senescent cells become more tissue destructive [53, 54]. Despite qualitative and quantitative differences of the SASP among different tissues and senescence models, a group of SASP factors including interleukin-6 (IL-6), CXC chemokine ligand 8 (CXCL8, or IL-8) and monocyte chemoattractant protein 1 (MCP1, or CCL2) can be expressed in over 50% of all types of senescent cells investigated [11, 18]. The SASP can comprise not only pro-inflammatory and pro-apoptotic molecules but also enzymes involved in ECM remodeling, such as various collagen proteins, matrix metalloproteases (MMPs), cysteine/serine protease suppressors (SERPINs), and tissue inhibitors of metalloproteases (TIMPs) [55–58]. The secretome of senescent cells can also reduce the expression of α-Klotho, a geroprotective protein that attenuates deleterious changes with aging and diseases, in multiple non-senescent human cell types, a process partially preventable by neutralizing antibodies against the SASP factors, activin A and IL-1α, further amplifying detrimental effects of senescent cells [59].

SASP factors are associated with multiple functions, with a subset of them being actively involved in chronic inflammation and contributing to diverse pathologies in the course of aging [60]. Some pro-inflammatory SASP components such as IL-1, IL-8, and tumor necrosis factor-alpha (TNF-α) can attract immune cell types including T cells, natural killer cells (NKs), dendritic cells (DCs), macrophages, and neutrophils [61]. As a result, senescent cells developing a pro-inflammatory SASP within various tissues and organs are subject to clearance by the immune system, contributing to the beneficial role of cellular senescence in the case of pathologies such as those involving tumorigenesis and tissue damage.

Senescent cells may not only develop a SASP and reinforce their own senescence-associated phenotypes, but induce a passive form of senescence of adjacent as well as distant cells that were originally normal or proliferating [40, 62, 63]. However, the SASP can confer resistance against immune clearance to senescent cells, as exemplified by the up-regulation of ligands such as human leukocyte antigen E, which can inhibit the function of CD8^+^ T and NK cells [64]. Under such conditions, accumulation of senescent cells may contribute to failed immune clearance, while allowing formation of an amplified SASP signaling cascade and causing the secondary induction of cellular senescence. Thus, senescent cell burden can be augmented by an aged or disabled immune system, since both innate and adaptive immune cells such as T cells with mitochondrial dysfunction can contribute to a global increase of senescent burden associated with physical frailty and multimorbidity in a process mediated by TNF-α signaling [65]. In such cases, the SASP can be responsible for an age-related inflammatory phenomenon termed “inflammageing,” which was first introduced by Franceschi, involves the chronic, low-grade sterile inflammation common across tissues in advanced age and appears prematurely in patients with cardiometabolic diseases [66–68]. Notably, blocking TNF-α signaling or preventing senescence with nicotinamide adenine dinucleotide (NAD) precursors partially rescues premature aging in mice with immune deficiency, such as those harboring T cells with dysfunctional mitochondria due to mitochondrial transcription factor A [65]. Thus, T cells are functionally involved in regulating organismal fitness and lifespan, highlighting the importance of tight immunometabolic control in both aging and the onset of age-associated diseases.

As the causative agent of the coronavirus disease 2019 (COVID-19) pandemic, human severe acute respiratory syndrome coronavirus 2 (SARS-CoV-2), causes upper respiratory infections and subsequently lung symptoms. Of note, new studies revealed that certain pathogen-related factors including lipopolysaccharide and SARS-CoV-2 spike protein-1 (S1), can markedly amplify the SASP extent of existing senescent cells, thereby generating increased risks for cytokine storm and related mortality in the elderly, and those with chronic pathologies associated with a high burden of senescent cells due to viral infections [53, 54]. Senescent cells can become hyperinflammatory upon exposure to pathogen-associated molecular patterns, up-regulating viral entry proteins and down-regulating antiviral factors in non-senescent cells through paracrine mechanisms. Thus, reducing the senescent cell burden in diseased or aged individuals holds the potential to enhance resilience and control mortality after infection, as is the case with SARS-CoV-2.

Supporting this, another study revealed that virus-induced senescence (VIS) is basically indistinguishable from other forms of cellular senescence and is also related to a SASP, while patients with COVID-19 display senescence markers in their airway mucosa *in situ* and enhanced levels of SASP factors in the serum [69]. *In vitro* data showed macrophage activation with a SASP-reminiscent secretory phenotype, complement lysis and SASP-escalating secondary senescence of endothelial cells, largely resembling hallmark properties of COVID-19 including macrophage and neutrophil infiltration, vascular endothelialitis, lymphocytopenia, and comprehensive thrombosis in affected lungs [70–72]. Furthermore, supernatant from VIS cells, including SARS-CoV-2-induced senescence, induced formation of neutrophil extracellular trapping and activation of platelets and the clotting cascade [69]. These findings establish VIS as a pathogenic trigger of COVID-19-related cytokine storm and organ damage, implying targeting of virus-infected cells, which are widely senescent and develop a SASP, as an ideal treatment option.

Furthermore, we recently reported that SARS-CoV-2 induces senescence in human non-senescent cells and amplifies the SASP in human senescent cells via Toll-like receptor 3 (TLR-3), which senses viral RNA and is up-regulated in human senescent compared to non-senescent cells [54]. Importantly, targeting TLR-3 through genetic or pharmacological approaches prevents senescence induction and SASP amplification by SARS-CoV-2 or Spike pseudotyped virus, supporting the proposal that clinical trials of senescent cell elimination (senolytics, reviewed below) and/or SASP/TLR-3-specific inhibitors to alleviate acute and long-term SARS-CoV-2 sequelae are highly warranted.

The SASP is one of the major contributors of chronic inflammation, a leading risk factor for the incidence of chronic pathologies such as neurodegenerative disorders, an example of which is Alzheimer’s disease (AD). With age, astrocytes become increasingly activated and display intensive secretion of inflammatory factors, while there is a significant overlap between reactive astrocyte-derived factors and the SASP components [73]. Elevated expression of SASP factor mRNA (IL-1β, IL-6, IL-8, and TNF-α) was observed in the hypothalamus of aged mice and in astrocytes induced senescent by estradiol *in vitro* [74]. Besides astrocytes, microglia can also become senescent and influence the pro-inflammatory state of the brain. For example, microglial cells derived from rat brain and cultured *in vitro* undergo stress-induced senescence, which is accompanied by the SASP [75]. Microglia of aged mice is characterized by higher granularity and increased expression of TNF-α, IL-1β, IL-6, cytokines that reflect development of the SASP [76]. Thus, the increasing inflammation in the central nervous system (CNS), or neuro-inflammation, can be tightly correlated with the accumulation of senescent cells, which represent a source of damaged macromolecules that generate pro-inflammatory responses in the CNS microenvironment, which represent a harmful condition that negatively influence CNS functioning. To the contrary, a whole body clearance of senescent cells can reduce activation of brain components such as the microglial cells, lower the SASP expression, thereby alleviating age-related brain inflammation and cognitive decline, a therapeutic efficacy demonstrated in aged *INK-ATTAC* mice with senescent cell eliminating agents [77].

## Strategies for targeting senescent cells

The detrimental effects of senescent cells in age-related pathologies can be profound, long-term, and comprehensive. Senescence causes an arrest of the cell cycle, including both differentiated and progenitor cells, and subsequent loss of regenerative capacity of tissues across multiple organs [78]. These nonproliferating and static senescent cells occupy key niches in the space of tissue microenvironments, blocking the proliferation of normal cells, and promoting tissue dysfunction, actions exerted via SASP-associated paracrine effects [42, 79, 80]. The contribution of senescent cells to age-related conditions, particularly frailty and multimorbidity, has been demonstrated by preclinical trials involving genetically engineered mouse models such as *INK-ATTAC*, *p16-3MR,* and PLD (from which highly p21-exressing senescent cells can be selectively eliminated) mice [81–84]. Experimental data from these models have increased our knowledge of whether senescent cells contribute to organismal aging and age-related pathologies. Most of these conditions were chronic diseases, such as AD, Parkinson’s disease (PD), atherosclerosis, chronic obstructive pulmonary disease, idiopathic pulmonary fibrosis, obesity/metabolic disease/insulin resistance, osteoporosis, and osteoarthritis [85]. To date, a number of therapeutic agents including but not limited to small molecule compounds have been reported with the capacity to specifically target senescent cells, representing a major advance in aging biology and geriatric medicine.

### Senomorphics

To date, some natural or synthetic agents have been reported to have therapeutic effects against senescent cells. These drugs, broadly termed as senotherapeutics, are generally classified into two major subcategories, namely senomorphics and senolytics, which pharmacologically target senescent cells by modulating the expression status of senescent cells or through inducing senescent cell death, respectively [80]. Such a distinction might be arbitrary, as compounds with senomorphic capacity in one cell type or context may have a senolytic effect in another, and vice versa.

Senomorphics prevent the expression of or antagonize the release of the SASP components, thus attenuating the deleterious consequences of those senescent cells with a pro-inflammatory, pro-apoptotic SASP, and reducing senescence-induced inflammation without killing senescent cells, although continuous administration of these agents is usually required. Mechanistic insights derived from senescence studies provide a molecular foundation for the use of SASP antagonists, such as the mTOR inhibitor rapamycin, to minimize the impact of senescent cells in pathological settings *in vivo*. Treatment with rapamycin (or its analogue, RAD001) diminished the SASP, disrupted senescence progression and mitigated liver dysfunction in naturally aged animals [36, 86–88]. More importantly, rapamycin treatment increased lifespan and delayed a number of age-related physical dysfunctions in mice [89].

As a critical mediator of SASP development, the transcriptional complex NF-κB can be targeted by chemical agents including metformin, apigenin, kaempferol, and BAY 11-7082 to abrogate the expression of SASP components [90–93]. NAD+/NADH metabolism has recently been identified as a critical regulator of SASP magnitude, and such regulation appears to be independent of senescence-associated growth arrest [94]. Neutralizing antibodies against key components of the SASP or their receptors, including IL-1α, IL-1β, IL-6, and TNF-α, can have senomorphic effects [85]. In addition, establishment of the SASP and secretion of SASP factors can be modulated by heat shock protein 90 (HSP90) inhibitors [95, 96].

Caloric restriction (CR), a therapeutic approach that can delay development of age-related dysfunction and increase lifespan, can minimize the activation of the mTOR signaling pathway while enhancing that of AMP-activated protein kinase (AMPK) [97, 98]. Several commercially available drugs targeting these molecules, including the mTOR inhibitor rapamycin and the AMPK activator metformin can prevent cellular senescence and dampen the SASP in experimental models [99, 100]. Alternatively, control of ROS accumulation with the ROS scavenger chemical *N*-acetyl-l-cysteine can markedly inhibit senescence and the SASP in human cells *in vitro* [101].

Employment of CR/dietary restriction (DR) mimetics may represent one promising and practical strategy for pharmacological targeting of senescent cells [98]. In humans, CR protects against cellular deterioration by reducing oxidative stress and minimizing cellular damage by enhancing the expression of genes encoding for HSPs and factors involved in autophagy [102, 103]. Moreover, CR down-regulates mTOR activity and hence restrains the SASP, an mTOR- and NF-κB-dependent pro-inflammatory phenotype of senescent cells [87, 104], underscoring the potential of CR to prevent the activation of primary and secondary senescence by suppressing mTOR machinery (Table 1).

**Table 1.** Senomorphic agents demonstrated by biomedical studies

Agent	Targets	Clinical trial status	References
Apigenin	NF-κB p65 subunit and IκB	Naturally available flavonoid	[92]
Metformin	IKK and/or NF-κB	Phase IV (NCT03708549) for metabolic syndrome in Schizophrenia patients Approved for type 2 diabetes	[91]
BAY 11-7082	NF-κB p65 subunit and IκB	Cell-based *in vitro* model of senescence	
Kaempferol	NF-κB p65 subunit and IκB	Naturally available flavonoid	[92]
SB203580	p38 MAPK	Cell-based *in vitro* model of senescence	[49]
(5*Z*)-7-Oxozeaenol	TAK1	Preclinical animal models	[36]
Rapamycin	mTOR	Phase II (NCT03359538) for amyotrophic lateral sclerosis Approved for immunosuppression	[86, 87]
RAD001	mTOR	Phase II (NCT00782626) for children with chemotherapy-refractory progressive or recurrent low-grade gliomas Phase II (NCT00449748) for patients with systemic mastocytosis Approved for tuberous sclerosis complex-associated diseases	[36]
Ruxolitinib	JAK	Phase II (NCT01950780) for alopecia areata (autoimmune disease) Approved for graft-versus-host disease	[48]
KU-60019	ATM	Preclinical animal models	[105]
Loperamide	HSP90	Phase III (NCT02008565) for fecal incontinence Approved for treatment of diarrhea	[95]
Simvastatin	IL-6, IL-8, and MCP1	Cell-based *in vitro* model of senescence	[106]
Cortisol	IL-6 secretion from senescent cells	Steroid hormone	[107]
Anakinra	IL-1R	Phase I (NCT01122914) for severe atopic dermatitis Phase II/III (NCT04443881) for cytokine storm syndrome secondary to COVID-19 Phase III (NCT04364009) for COVID-19 respiratory symptoms Phase IV (NCT02219828) forrecurrent idiopathic pericarditis Approved for rheumatoid arthritis	[108]
Canakinumab	IL-1β	Phase I (NCT03936894) for Duchenne muscular dystrophy Phase III (NCT04717635) for adult onset Still’s disease Phase III (NCT00770601) for neonatal-onset multisystem inflammatory disease Approved for cryopyrin-associated periodic Syndromes	[109]
Etanercept	TNF-α	Phase I (NCT00107991) for treatment of Hidradenitis Phase II (NCT00329823) for therapy of hydradenitis suppurativa Phase IV (NCT00873730) forankylosing spondylitis Approved for autoimmune diseases	[110]
Siltuximab	IL-6	Phase I/II (NCT02796859) for schizophrenia and other psychotic disorders Phase II (NCT04975555) for cytokine release syndrome (CRS) and Immune effector cell associated neurotoxicity Approved for multicentric Castleman disease	[111]
Tocilizumab	IL-6R	Phase not available (NCT04924829) for severe COVID-19 pneumonia Phase II (NCT04332094) for SARS-CoV-2 infection Phase IV (NCT04730323) for COVID-19 associated cytokine release syndrome Phase IV (NCT03781310) for rheumatoid arthritis Approved for autoimmune diseases	[112]

### Senolytics

The class of senolytics has largely evolved from early studies demonstrating the feasibility of senescent cell elimination with combined use of the Src kinase inhibitor dasatinib and the plant flavonoid quercetin (hereafter, “D + Q”) [113]. Although the discovery of senolytics was through a hypothesis-driven, mechanism-based and bioinformatics-guided approach to screen agents with the potential to disrupt the senescent cell anti-apoptotic pathways (SCAPs) and other pro-survival networks [113], the class later expanded to employ additional senescence features and enhance clearance of senescent cells by the immune system. The first generation of senolytic agents can transiently disable SCAPs, and allow the tissue-damaging SASP of senescent cells to kill themselves. In contrast to the one-drug, one-target mechanism, SCAP interference may perturb a group of pro-survival pathways simultaneously [80]. Hence, these early senolytics typically act through several pharmacologic mechanisms and achieve therapeutic effects synergistically. For instance, the “D + Q” combination exerts a wide-spectrum senolytic activity by interfering with a few pro-survival networks, including ephrin dependence receptor signaling, PI3K-Akt and BCL-2 molecules. Of note, BCL-2 family members including Bcl-2, Bcl-_X_L, and Bcl-w, which prevent activation of pro-apoptotic signaling cascades, cytochrome *c* release from mitochondria and downstream caspase activation in the cytoplasm, are targeted alternatively by other senolytics. Fisetin, a plant flavonoid polyphenol, is another senolytic agent, but with hitherto-unknown mechanisms of action (as is the case for most of these agents) [114], although it can enhance the expression of antioxidant PON2 via activation of PPAR*γ* in a dose-dependent manner to efficiently alleviate neointimal hyperplasia after intimal injury [115].

While all senolytic strategies may cause off-target effects or interfere with beneficial cell subpopulations, these actions are generally limited as most treatments can be administered as intermittent “hit-and-run” dosing modalities, which do not rely on daily or weekly administration [11]. In particular, such intermittent dosing strategies may be efficacious since it takes senescent cells ~7 days or more to become established (at least *in vitro*) and to develop a pro-inflammatory and pro-apoptotic SASP after exposure to damaging insults [38, 116], and it takes even longer for these cells to re-accumulate.

Small molecule inhibitors of Bcl-2/Bcl-xL, such as ABT-263 (navitoclax), A1331852, and A1155463, are senolytic against senescent human lung fibroblasts and umbilical vein endothelial cells (HUVECs) [113, 114, 117]. However, in contrast to “D + Q,” these agents cannot broadly eliminate senescent populations *in vitro*, and resistance to them has been shown in senescent preadipocytes, which can induce metabolic dysfunction and be harmful *in vivo* [118]. As a major drawback, moreover, the senolytic potential of Bcl-2/BcL-xL inhibitors is limited by their off-target effects on neutrophils, platelets, and potentially T cells, which might compromise hemostasis and further impair immune function [119–121]. Additional SCAPs that can be drug-targeted involve modulation of p53-associated pathways and interruption of the anti-apoptotic transcription factor FOXO4, suppression of the peptidase USP7 and perturbation of HSP90 chaperones [95, 122, 123].

As a distinct strategy for inducing senescent cell death, the enhanced activity or increased expression of specific molecules has been ingeniously exploited. Nanoparticles conjugated with either fluorophores or toxic agents and coated with galacto-oligosaccharides can preferentially deliver a cytotoxic cargo to senescent cells, mainly taking advantage of the higher level of SA-β-Gal activity in these cells [124]. Recent studies have substantiated the rationale of delivering cytotoxic chemicals to lysosomes of senescent cells through galactose-modifying prodrugs or related agents [125, 126].

Ouabain, a cardiac glycoside, has a remarkable senolytic capacity with the broad activity against different types of senescent cells [127]. Specifically, ouabain induces apoptosis partly by mediating up-regulation of the pro-apoptotic BCL-2 family member NOXA in senescent cells. Furthermore, cardiac glycosides can synergize with anticancer drugs to kill cancer cells and deplete senescent cells that tend to accumulate after irradiation or in old animals. Procyanidin C1 (PCC1), a natural polyphenolic component of grape seed extract, holds the potential to increase both healthspan and lifespan of experimental mice by targeting senescent cells [128]. Interestingly, PCC1 appears to be a dual-purpose agent, as it inhibits SASP formation at low concentrations, while selectively eliminating senescent cells at higher concentrations, the latter through enhancing ROS production and inducing mitochondrial dysfunction. In rodents, PCC1 clears senescent cells in the treatment-damaged tumor microenvironment and increases therapeutic efficacy upon co-administration with chemotherapeutic drugs. Of note, intermittent administration of PCC1 to irradiated, senescent cell-transplanted or naturally aging mice considerably alleviates physical dysfunction and increases overall survival (Table 2).

**Table 2.** Senolytic agents reported by literatures

Agent	Targets	Clinical trial status	References
Dasatinib	Pan-receptor tyrosine kinases including ephrin B1	Phase II, (NCT02848131) for chronic kidney disease Phase II (NCT04313634) for skeletal health in older humans Phase I/II (NCT04063124) modulate progression of AD (SToMP-AD) Phase not available (NCT02652052) for premature aging and senescence in hematopoietic stem cell transplant survivors	[113]
Quercetin	PI3K and many others	Phase II, (NCT02848131) for chronic kidney disease Phase II (NCT04313634) for skeletal health in older humans Phase I/II (NCT04063124) modulate progression of AD (SToMP-AD) Phase not available (NCT02652052) for premature aging and senescence in hematopoietic stem cell transplant survivors	[113]
Fisetin	PI3K/AKT/mTOR	Phase I/II (NCT04210986) for osteoarthritis-related articular cartilage degeneration Phase II (NCT04313634) for skeletal health in older humans Phase I/II (NCT04815902) to improve the effect of bone marrow stem cells for steoarthritis Phase II (NCT04476953) to alleviate complications of SARS-CoV-2 Phase II (NCT03675724) for frailty, inflammation, and related measures in older adults Phase II (NCT03430037) for frailty, inflammation, and related measures in older women Phase II (NCT03675724) for frailty, inflammation, and related measures in older adults Phase II (NCT03325322) for Inflammation and Stem Cells in Diabetic and Chronic Kidney Disease	[114]
PCC1	NOXA/PUMA/mitochondrial membrane potential/ROS	Preclinical animal models	[129]
ABT-263 (navitoclax)	Bcl-2, Bcl-_X_L, and Bcl-w	Phase I/II (NCT00481091) Phase I/II (NCT02079740) Phase I/II (NCT00406809) Phase I (NCT00982566) Phase I (NCT00743028), Phase I (NCT00888108) for various cancer types	[130]
ABT-737	Bcl-2, Bcl-_X_L, and Bcl-w	Preclinical animal models Phase not available (NCT01440504) for ovarian tumors (*ex vivo*)	[131]
A1331852	Bcl-_X_L	Cell-based *in vitro* models of senescence	[114]
A1155463	Bcl-_X_L	Cell-based *in vitro* models of senescence	[114]
Cardiac glycosides including oubain and digoxin	Bcl-2, Bcl-_X_L, and Bcl-w	Preclinical animal models	[127, 132]
EF24	BCL-2 family members	Cell-based *in vitro* models of senescence	[133]
UBX0101	MDM2 and p32	Phase II (NCT04129944) for osteoarthritis of the Knee	[82]
Azithromycin	Aerobic glycolysis and autophagy	Cell-based *in vitro* models of senescence	[134]
Roxithromycin	Aerobic glycolysis and autophagy	Cell-based *in vitro* models of senescence	[134]
Proxifim	Pro-ferroptotic lipoxygenase-5 and antiferroptotic glutathione peroxidase 4 (GPX4)	Preclinical animal models	[135]
Geldanamycin	HSP90	Cell-based *in vitro* models of senescence	[95, 136]
Alevspimycin (17-DMAG)	HSP90	Cell-based *in vitro* models of senescence	[95]
Piperlongumine and analogues	OXR1 and others	Cell-based *in vitro* models of senescence	[137]
Galactose-conjugated nanoparticles	Lysosomal activity of senescent cells	Preclincal animal models	[124]
Galactose-modified cytotoxic agents	Lysosomal activity of senescent cells	Preclinical animal models	[125]
Ferroptosis inducer RSL3	Lipoxygenase-5, glutathione peroxidase 4	Preclinical animal models	[135]
Senescence-specific killing compound 1 (SSK1)	Lysosomal β-galactosidase	Preclinical animal models	[138]

Despite these advances, there are a few technical caveats. ABT-263 eliminates senescent fibroblasts and HUVECs, but exhibits little effects on human pre-adipocytes [130, 139]. The combinatorial format of “D + Q” induces apoptosis of all these three types of senescent cells in a dose-dependent manner, but is frequently toxic to non-senescent cells [113, 122, 140]. The natural flavonoid fisetin has been reported as a potential senotherapeutic agent, but shows a modest efficacy in eliminating senescent fibroblasts and pre-adipocytes even at high concentrations [114]. The galactose-modifying prodrug, senescence-specific killing compound 1 (SSK1), which can be specifically activated by β-galactosidase and employed to eliminate mouse and human senescent cells, has the risk of targeting non-senescent cells, such as macrophages which are generally SA-β-Gal-positive upon activation by acute inflammation or cytokine storm, thus additional caution should be exercised upon application to target senescent cells *in vivo* [138].

## Preclinical trials of senolytic agents

### Alleviation of multiple conditions in experimental mouse models

To date, a handful of senolytics have been examined in a variety of preclinical models. In naturally aged mice and atherosclerosis mouse models, “D + Q” administration improved cardiac function [141]. In radiation-damaged mice, “D + Q” enhanced exercise capacity [139]. Ercc1(-/Δ) mice accumulate spontaneously occurring endogenous DNA damage and model a human progeroid syndrome, while “D + Q” delayed the onset of several age-related symptoms including physical dysfunction in them [95]. In osteoporosis mouse models, administration of “D + Q” increased bone mass and strength, providing a novel treatment strategy for not only osteoporosis but multiple age-related comorbidities [142]. Adipose tissue inflammation and dysfunction are causative for obesity-related diabetes and insulin resistance, but “D + Q” alleviated metabolic and adipose tissue dysfunction in obese mice, suggesting the potential of senolytics in treating obesity-related metabolic dysfunction and complications [83]. In bleomycin-treated mice that develop idiopathic pulmonary fibrosis and accumulate senescent cells in lung tissues, “D + Q” depleted senescent fibroblasts and improved physical and pulmonary function, although lung fibrosis remained mainly unaltered [140].

Fisetin has also generated favorable preclinical data in recent studies. For example, fisetin triggered substantial death of senescent HUVECs by inducing apoptosis [140]. Furthermore, fisetin attenuated age-related pathologies and increased overall lifespan in wild type mice [143]. A recent study documented the efficacy of fisetin in reducing the symptoms of various neurodegenerative disorders, including amyotrophic lateral sclerosis, AD, PD, Huntington’s disease, and traumatic brain injury [144]. With the growing body of preclinical data, along with fisetin’s capacity to interfere with a large array of pathways associated with brain dysfunction, there is an increasing interest in pursuing the therapeutic effects of this natural flavonoid in humans.

Senolytic agents have exhibited substantial efficacy in delaying, preventing or alleviating physical frailty, multiple cancers, and a variety of cardiovascular, liver, kidney, musculoskeletal, lung, eye, hematological, metabolic, and neuropsychiatric pathologies as well as complications of cancer treatment and organ transplantation [13]. Through targeting fundamental aging mechanisms, which are considered as “root cause” contributors to multiple chronic disorders, senolytics hold the potential to alleviate over 40 or even more age-related conditions as illustrated by preclinical studies, thus opening a new avenue for treatment of age-related dysfunction and chronic pathologies.

### Translation of senolytics into interventions for humans

Given the promising effectiveness of senolytics for a host of different disease models as supported by recent preclinical data, these agents have been keenly considered for translation via clinical trials. Due to the novel nature of senolytic agents and their unknown risks, senolytics were first tested in patients with serious conditions including diabetes, IPF and osteoporosis [13, 15]. Fisetin and quercetin, two naturally derived flavonoids, displayed favorable safety profiles and were chosen for a variety of clinical trials, some of which also recruit healthy aged adults. Dasatinib has been in clinical use since 2006, with a well-understood safety profile. With approval from the Food and Drug Administration (FDA), the first wave of clinical trials involving “D + Q” and fisetin has been launched, while many more are planned.

## Clinical trials: active and planned

Data from early pilot trials indicated that senolytics potently eliminate senescent cells, alleviate inflammation and attenuate frailty in humans. Clinical trials for AD, COVID-19, diabetes, eye disorders, IPF, osteoarthritis, osteoporosis, bone marrow transplant, and childhood cancer survivors are underway or just beginning. Caveats are that until such studies are done, it is too early for senolytics to be applied for the aged outside of clinical trials.

### Active trials, completed trials, and early results

The first clinical trial of senolytics demonstrated improved physical function in IPF patients after “D + Q” administration in a first-in-human, open-label, and pilot study, warranting evaluation of “D + Q” in expanded randomized controlled trials for senescence-related disorders [145]. Similarly, another pilot clinical trial reported that treatment with “D + Q” reduces senescent cell burden in adipose tissues of patients developing diabetic kidney disease [146]. Specifically, adipose tissue activated macrophages and adipose tissue fibrosis (crown-like structures) were decreased, as were levels of circulating SASP factors, including IL-1α, IL-6, and MMPs, confirming target engagement (senescent cell decreases) using a “hit-and-run” treatment strategy with senolytics. More recently, an open-label early phase 1 trial of “D + Q” for AD (SToMP-AD) reported that intermittent senolytic administration decreases tau protein accumulation and neuro-inflammation, preserves neuronal and synaptic density, partly restores cerebral blood flow and reduces ventricular enlargement [147]. The results support the initiation of a randomized, double-blind and placebo-controlled multicenter phase II trial, which aims to further explore the safety, feasibility, and efficacy of senolytics in treating AD. Currently active trials with senolytics include, but are not limited to, those listed in Table 2.

### Planned trials in schedule

Given the relative safety and remarkable efficacy of senolytics demonstrated by the first clinical trials [145, 146], a number of new trials that investigate the clinical profiles of senolytics have been initiated or planned. Strikingly, the senolytics including fisetin and “D + Q” proved effective for treatment of a mouse β-coronavirus related to SARS-CoV-2 in mice [53]. In light of the current COVID-19 pandemic worldwide, several trials for COVID-19 intervention are underway, among which there is an ongoing trial for hospitalized patients (COVID-FISETIN: Pilot in SARS-CoV-2 of Fisetin to Alleviate Dysfunction and Inflammation, NCT04476953).

## Translational Geroscience Network

To support continued and advanced research of senolytic agents, a US National Institutes of Health-funded Translational Geroscience Network (TGN) has recently been established to conduct clinical trials with the aim of targeting different fundamental aging-associated mechanisms in parallel. The TGN comprises a group of scientists and clinicians from different institutions including Mayo Clinic, Connecticut, Harvard, John Hopkins, Wake Forest, the Universities of Minnesota and Michigan, and the University of Texas Health Sciences Center at San Antonio, with other partners at St. Jude Children’s Cancer Hospital, City of Hope, Northwestern University, the Steadman Clinic, and other sites.

The TGN Facility for Geroscience Analysis (FGA) is a laboratory core that develops and performs innovative assays for fundamental aging process and pathology-specific markers across the trials [15]. As these tests are currently being conducted to evaluate target engagement, efficacy of senolytics, and other Geroscience interventions through preclinical and early phase clinical trials, the FGA is exploring new markers of basic aging mechanisms and age-related disorders.

## Research limitations

Through conducting pilot trials across various age-related conditions and using different types of senolytic agents, the Geroscience Hypothesis can be tested in human patients. This hypothesis postulates that by targeting a fundamental aging process or a hallmark of biological aging, such as cellular senescence, multiple age-related pathologies can be remarkably delayed, alleviated or effectively prevented, as a group, instead of one-at-a-time [148]. By definition, the aim of developing senolytics is to target senescent cells, while the long-term goal is to restrain development of geriatric diseases, extend human healthspan, and reduce morbidity toward the end of life. To date, it seems not feasible to identify a single molecular target, or to develop a drug that affects that single target, specifically in the pipeline of senolytics. Currently available senolytics could cause apoptosis in multiple cell types, including non-senescent cells (generating systemic effects), or hamper elimination of a small subset of senescent cells expressing a certain target. Although we show that senolytics such as “D + Q” is effective in diminishing senescent cells in current clinical trials, with several first lines of direct evidence supporting the effectiveness of senolytics in humans, there is still much to be done yet. If clinical trials over next few years substantiate and extend our findings to demonstrate that these agents can alleviate additional age-related disorders (beyond IPF, diabetic kidney disease, and AD) by reducing senescent cell burden, senolytics might become an entirely new therapeutic modality for intervening currently untreatable chronic diseases and increasing human healthspan.

## Concluding remarks and future prospects

The hallmarks of aging, particularly cellular senescence, contribute to age-related pathologies, and physical dysfunction. The Unitary Theory of Fundamental Aging Mechanisms highlights the interdependence between the hallmarks of aging and suggests interventions targeting one fundamental aging hallmark may impact many or all of the others. Accumulation of senescent cells is correlated with and responsible for cardiovascular diseases, frailty, obesity, cognitive decline, diabetes, and other age-and/or chronic pathology-related conditions, implicating senescent cells as a potential and competent target for interventions. So far, mounting lines of evidence support the efficacy of senescence-targeting agents, namely senotherapeutics, which mainly comprise senomorphics and senolytics. Early preclinical data about senolytics, small molecule agents that eradicate senescent cells, have shown promising indications of effectiveness across several aging and disease models. The first wave of in-human trials with the senolytic combination of dasatinib and quercetin suggested decreased senescent cell burden in adipose tissue of patients with diabetic kidney disease and improved physical function in patients with idiopathic pulmonary fibrosis. Clinical trials with other senolytics, including the flavonoid fisetin and Bcl-xL inhibitors, are currently in progress. Although exciting results derived from preclinical and early clinical trials suggest there is potential for senolytics to alleviate age-related physical dysfunction and pathologies, more and continued clinical trials across different aging and disease models are essential and desperately needed. As the global population suffering from age-related health conditions continues to expand, it is important to establish collaborations and infrastructures like the TGN to orchestrate parallel trials across institutions and accelerate translation of senolytic agents, including both established agents and those newly emerging, into future clinical practice.
